# American Nervousness—Its Philosophy and Treatment

**Published:** 1879-09

**Authors:** George M. Beard

**Affiliations:** New York.


### ARTICLE III.

[*Continued from August Number.*]

### *American Nervousness: Its Philosophy and Treatment.*

BY GEORGE M. BEARD, M. D., NEW YORK.

An Address delivered before the Baltimore Medical and Surgical Society, February 12th, 1879.

What, now, are the causes of this increase of nervousness in America during the past half century? There is no single cause; it is a combination of influences that have brought about this unparallelled condition of the nervous styst m. The primary cause is unquestionably civilization, especially with its recent accompaniments, as the telegraph, railway and the periodical press. These three institutions have drawn, and continue to draw each year, most severely on the nerves of nearly all classes; but particularly upon those who are favored with education. The introduction and popularization of the railway and the telegraph, and the development of the periodical press, belong, it will be observed, to the nineteenth century; and they have intensified in ten thousand ways cerebral activity and worry. This factor of civilization applies to all the great countries —Europe as well as America.

But after we have given this cause every credit to which it is entitled, we are yet face to face with this question. Why are the Americans more nervous than any other people on this planet? The answer to this question, which has occupied the thoughts of philosophical observers for the past quarter of a century, is to be found mainly in these factors: First, the dryness of our atmosphere; and secondly, the extremes of heat and cold. In these two respects, America differs from any other civilized country.

Dryness of atmosphere produces nervousness in two ways: first, by taking up and absorbing the moisture of the body, thus causing us to literally dry up. When the atmosphere is moist, perspiration accumulates upon the surface of the body, because the air cannot take it up. Hence, in our dull dog-days, we are frequently annoyed by excessive perspiration. In a dry air which is hungry for moisture, the fluids of the body, as they become vaporized, are rapidly conducted away; the body is thus wasted of its fluids. Dry air also prevents the electricity of the body from being conducted away, and thus we become excessively charged with that force, and excessively stimulated by its confinement in the body. Moisture conducts electricity; and the moistened air intensibly carries away the electricity of the body, so that it is impossible for the body to become so excessively charged and stimulated. The evidences of this dryness of our atmosphere are numerous and striking. Clothes on the line dry more rapidly than in Europe. The specimens of the naturalists do not so quickly mould; the hair is stiffer and dryer than that of our European contemporaries, and requires more pomade and oil. This peculiarity of our climate is observed from the Atlantic to California; and the Rocky Mountain region is far more under the influence of this dryness of atmosphere than even the East. In the region of northern Dakotah and Montana, as all authorities agree, buffalo and meats of other kind dry more speedily when exposed to the air, and keep much longer than in other countries. The electrical state of what I have spoken, and the charged condition of the body, is also very commonly observed in that section of the country. A comb drawn through the hair produces crackling, and sparks come from the clothing while dressing and undressing in a dark room; and in some cases on the mountain sides, actual lightning is seen coming out of the rocks or soil. In this section of the country, also, where the air is so extremely dry, nervous people frequently become more nervous; they are troubled with insomnia and neuralgia, and various forms of debility. In the valley of Sacramento, as is well known, the north wind coming down from the mountains is exceedingly dry, somewhat like the simooms of the East; so that the whole earth—the metals upon it—become surcharged by the electricity which cannot escape; and the fruit on the side from which the wind is blown, becomes parched and shriveled, and the grass everywhere has its vitality impaired, while men and animals of all kinds become fretful and irritable. This extreme condition illustrates the process which is going on, though in a less degree, all over the northern and eastern portion of the United States. The violent extremes of heat and cold—the bitterness of our winters contrasted with the heat of our summers—excite nervousness by over stimulation. The application of latent heat and cold, as ice in hot water, is one of the most powerful means of local stimulation that we have in medicine; to this treatment, nearly all of the American people in the northern and eastern sections are constantly subjected.

Secondly, extreme heat and cold produce nervousness by compelling us to live in-doors in unnaturally dry and over-heated atmospheres, and making it impossible, either in summer or winter, to partake of those active out-door exercises and amusements in which our English friends indulge at nearly all seasons of the year. The English climate, as contrasted with the American, is more equable. Its moisture, and even its unpleasantness and disadvantageousness is favorable to the nervous system; likewise, the climate of our Southern States is more moist and more uniform than of the North and West: and, according to investigations that are variously made, nervous diseases of all kinds, or nearly all kinds, pretty steadily diminish in frequency as we go South.

The institutions of civilization common to all enlightened countries, such as schools, newspapers, excitement of elections, reforms and revivals, are themselves the results of climate and race, and are also to be included among causes of nervousness. Civilization is burdened with information that it must acquire; every year history raises up new facts, that the school-boy of the future must commit and recite. If we would know why the Americans are so nervous, we should contrast the Greek boy with the New York boy in their manner of training in the schools, in their play, and in the whole order of their lives. The Greek boy's life was a poem, a constant holiday, a perpetual picnic. Of study, toil or work, to which the New York boy is early trained, he knew nothing. Work is really a modern institution. All culture, history, science, literature and languages that have appeared in the world during the past two thousand years, the lad of to-day must try and acquaint himself with. Of all these, the Athenians knew nothing—

could not even predict. When we contrast the life of an American child, from its early school days until the hour it leaves the university or seminary, the many and tiresome hours of study, the endless committing and repeating and forgetting, the confinement in constrained positions, the over-heated and over-dried atmosphere, the newspapers and novels that he is and must be prepared to converse about and criticise: the sermons and lectures which he is compelled to listen to and analyse, the strife and struggle for bread and competence against inordinate competition, the worry and concentration of work made both possible and necessary by the railway, mail service and the telegraph; in view of these facts, we wonder not that the Americans are so nervous, but rather wonder at the power of adaptation of the human frame for unfavorable environment. The education of the Athenian boy consisted in play and games and songs, and repetitions of poems, and physical feats in the open air. His life was a long vacation, in which, as a rule, he rarely toiled as hard as the American lad in the intervals of his studies.

The rapidity of our modern and American life has a tendency to concentrate an enormous amount of activity in a brief space of time. The intensity, the fierceness and violence of our toil, are the results of our climate; and in their turn, they deepen and intensify our nervous sensibility. In the study of this subject, the disposition has been to look exclusively at some one of these secondary elements—our haste in motion or our haste in eating, and to consider some one such factor as the sole cause of American nervousness. Indeed, I may say, that up to the present time, this has been the popular mode of interpreting the unparallelled phenomena connected with American nervousness. Effects have, indeed, been confounded with causes—a process of reasoning which, it may be added, vitiates and destroys nearly all human philosophy, and nearly on all themes, but especially, on questions of sociology, such as the effects of stimulants and narcotics, or diet, or social customs. American nervousness is a complex resultant of a number of factors—not a single result of one. In order to understand it, to grasp it, to master its philosophy, we must be able to see these factors all at once by themselves, and in their relations to each other.

There is one disease, the type and centre of a large family of functional diseases, to which I have applied the term *neurasthenia.* If we understand the philosophy of this disease and its treatment, there will be little difficulty in understanding the philosophy and treatment of very many of the family of functional nervous diseases to which it belongs. Neurasthenia is pre-eminently an American disease. It might, indeed, be properly called *Neurasthenia Americanna.* Although it is found in England and on the Continent, it was here first systematically described, and here it exists in greater variety and frequency than in all other countries combined. The generic term neurasthenia —nervous exhaustion—I subdivide into two: cerebrasthenia —exhaustion of the brain; myelasthenia—exhaustion of the spinal cord.

Among the symptoms that I have referred to *cerebrasthenia* (brain exhaustion) are tenderness of the scalp, cerebral irritation, tenderness and whiteness of the teeth and gums, flushing of the face, special idiosyncrasies in regard to food and external irritants, morbid desire for stimulants and narcotics, insomnia in its varied manifestations, dilated pupils, melancholia or mental depression, deficient memory, or power of intellectual control, different forms of morbid fear, as astruphobia (fear of lightning,) agoraphobia (fear of places,) anthropophobia (fear of man and society,) and its opposite, monophobia (fear of solitude,) sick headache, and various forms of headache, and pains in the head, disturbances of the nerves of special sense, as *tinnitus aurium,* and specks before the eyes, subjective tastes and odors, dryness of the skin, eyes, throat and mucous membranes generally. Among the leading symptoms of myelasthenia (exhaustion of the spinal cord,) are spinal irritation with general hyperæsthesia, irritation of the tip of the spine, coccogynia, irritable breast, irritable ovaries, irritable womb, vague pains throughout the body, flying neuralgias, tremulous and variable pulse, sometimes very high and sometimes very low, with occasional attacks of palpitations, sudden giving away of special or general functions, shooting pains in the limbs similar to those of ataxy, sudden starting on dropping to sleep, abnormalities of the secretions and dryness of the skin, or the opposite, excessive perspiration, local and general, as sweating of the hands and feet, gaping, yawning, stretching, neurasthenic forms of chilliness, creeping sensations in the spine, ticklishness, local spasms of muscles—the so-called fibrillary contractions.

This differentiation of the symptoms of cerebrasthenia and myelasthenia is not absolute; a number of these symptoms would seem to be common to both forms, and some, very likely, are due to disorders of the sympathetic in the cerebral or spinal region, or both. In some patients we have almost pure myelasthenia; in others, almost pure cerebrasthenia; in others, a combination, or, as is very frequently the case, an alternation of the two. Some of these symptoms, when they occur, themselves act as causes for other symptoms; for the sensitive nervous human body is like certain mountainous regions—full of echoes and reverberations. An irritation at one point may be transferred to any other point, following along the paths of least resistance, and making itself felt in those parts that are least able to resist molecular disturbanees. Thus, for example, seminal emissions and spermatorrhœa, when they arise through abuse or through spinal cord disease, almost uniformly react on the brain—robbing the sufferer of courage and manliness, exciting various phases of morbid fear, of which I have spoken, with aversion of the eyes and countenance. The reaction is also on the vocal apparatus, causing a neurasthenic voice; on the eyes bringing on neurasthenic asthenopia, against which glasses and gymnastics are powerless; on the stomach, inducing irritative dyspepsia ; on the sweet-centres, producing morbid sweating of the hands—palmar hyperdrosis ; on the face and hands, and ears especially, producing morbid flushing and redness. Likewise, also, nervous dyspepsia, whether excited directly by abuse of the stomach or by over-use of the brain, reacts on the brain itself and on the whole organism ; so that, an attack of indigestion is sometimes indicated by pains in the limbs, or by nervousness of a vague and indistinct character in different parts of the body.

It is of supreme scientific and practical importance to be able to make a differential diagnosis between the symptoms of neurasthenia in its different forms and the symptoms of early stages of grave structural lesions of the brain, spinal cord, peripheral nerves. To make such a differential diagnosis is sometimes the severest test to which the neurologist can be brought, and one of the highest value for the happiness, the plans, and the whole future of his patient. The not being able to meet this test has been, and is now, in all countries as well as our own—particularly in the last twenty-five years—a cause of frequent errors in the advice—both hygienic and medical—given to patients; for the prognosis and treatment of neurasthenia is oftentimes quite the opposite of the prognosis and treatment of incurable cerebral, spinal or peripheral nerve lesions. If we were compelled to be guided by isolated symptoms, it would be impossible, in many instances, for human skill to make such differential diagnosis between neurasthenia and some of the diseases that it stimulates ; for the symptoms, considered by themselves, are sometimes precisely the same; and of themselves alone would not point towards the solution of the problem. The tendency of neuropathology is not toward, but away from, the idea of single pathognomonic symptoms. It is by considering groups of symptoms in their relation to each other, and to the history of the case, that we make out, in recent times, the diagnosis of ataxia, or of any of the different forms of spinal disease, or of hay fever. Whenever any of the different phases of professional cromps develop, such as of musicians, or writers, or painters, or telegraphers, or designers, or engravers, or artists, or barbers, or counters of money, there are single symptoms in any one of these diseases, that in themselves, might mean rheumatism or neuralgia, or neuritis, or diseases of the joints or spine; and very often, indeed, this mistake in diagnosis is made in spite of all the literature and teachings upon this subject.

Neurasthenia is differentiated from organic disease, by taking into consideration these four elements: (1) The fluctuations and inconsistency of the symptoms; (2) heightened reflex action; (3) the existence of some certain special symptoms, which will rarely be found in organic spinal disease—such, for example, as different forms of morbid fears which I have described, palmar hyperidrosis, excessive tenderness of the spinal cord, deficient thirst, abnormally active pupils, mental depression, extreme insomnia, morbid desire for stimulants and narcotics. In certain organic diseases, it is true, there may be heightened reflex action; but, as a rule, reflex action is diminished in organic or structural disease of the spinal cord. Closely analyzed, a large proportion of the symptoms of neurasthenia, as I have before described them, are of a reflex character coming from the stomach, or some part of the genital apparatus, or, if they are not reflex in their origin, are at least made worse by a reflex irritation. To know this fact, and to act upon it in the treatment of these cases, is indispensable for success. One may treat sweating hands and flushing face and various neuralgias and headache indifferently without any permanent effect, until we attack and destroy the cause, which is often found in some portion of the genital apparatus. (4) Those in whom the nervous diathesia predominates, are likely to have functional nervous diseases.

In regard to the prognosis in cases of this kind, this general statement is sustained by experience, viz.: All of these cases can be relieved; many of them can be absolutely or approximately cured; but in all cases time and patience are necessary to bring about these results. I have watched these cases for years after they have left off treatment, and I keep up correspondence with patients who have been under my care, and thus have an opportunity to know what the issue is. Patients of the kind live to a good old age—may attain even unusual longevity, and may have their best health during the latter part of their lives.

Our "sick headaches" almost always leave us between 50 and 60, but of course, sometimes earlier—about 40 or before; and in this respect, sick headache is, I am persuaded, a type of a large number of functional nervous disturbances.

The results of treatment depend greatly on hygiene and therapeutics. These cases respond to treatment: they yield directly and positively to the influences of remedies. It has been the usual plan to manage cases of this sort, without making any special diagnosis, by what is called moral treatment, which, when scientifically employed, is mental therapeutics. This is a just and most potent means for controlling the disease, but is not to be used exclusively in these diseases any more than in any other disease; subjective treatment is merely an additional means of relief. In regard to the details of treatment, I will state but a few facts.

First comes *electricity* in its various modes of application—central, general and local. In the dosage, we are, in recent years, learning these four facts: 1. That it is sometimes best to use it in exceedingly small doses, mild currents and short applications. 2. That it is sometimes well to use a very strong and painful current. 3. That applications may be protracted for hours in succession. 4. That applications may be made much more frequently than is the general custom. These four propositions are not brought out in our text-books. I have not myself insisted upon them, as I mean to do hereafter when I refer to this subject. They have been impressed upon my mind especially of late, and are the direct results of experience with cases. These four propositions apply to nearly all our remedies. In truth, we are widening and deepening the system and range of our therapeutics forces by modification of the quantity and quality and mode of administration. I have long taught that for spasmodic difficulties, like local sprains, of muscles, convulsive tic, facial spasms, etc., very mild galvanic currents are preferable; but I have lately seen a case where very powerful and painful faradic currents, applied with the electric brush, or with the sponge, or both, and with as strong currents as could be borne, were more efficacious than the mild currents. Likewise in sciatica, and even other forms of neuralgia, painful currents that make a blister, or at least very irritating to the skin, may succeed after mild applications have failed. An electro-puncture directly into the nerve itself will cure when the mild currents are powerless. In the treatment of a new case, and until we have learned the temperament of the patient, and the way he responds to electricity, it is proper always to employ mild currents, and for the same reason that it is always best to begin with a minimum dose of any remedy. But, when necessary, it is also well to test the full physiological effects of the remedy before giving up a case. I am convinced that in many cases electricity will not give extraordinary effects until we have produced the physiological effects upon the patient, such as soreness of the muscles, nervous irritation, sleeplessness, temporary accerbation of symptoms and the like. To begin treatment with the excitation of these symptoms, is unwise as a rule; very many persons are over-galvanized and over-faradized. Every case in this respect must be itself a study. I formerly believed that an application once a day was, to say the least, enough; but I know from experience that applications twice a day, and, in some cases, applications quite prolonged are advantageous.

Many years ago I pointed out the fact that there are certain temperaments that do not bear electricity, or bear it very badly, and must be treated with mild currents, and with quite long intervals between the applications. Any new cases that come under our care may, for all we know, have this temperament. These propositions apply to all the other remedies of which I am to speak.

The ancients, you know, classed the divinities as major, minor—*Dii majores*, *Dii minores*. Similarly, neurotics m· y be divided into major and minor remedies. At the head of the major remedies—the *Jupiter omnipotence*—stands without question, electricity; then come ergot, the bromides, arsenic, strychnine, oils and fats, counter irritation, cannebis indica, massage or systematized manipulations of the muscles, the use of dry heat and cold, hydro-therapeutics, the zinc preparations, belladonna, digitalis and iron.

Of these various remedies, *ergot* is especially worthy of note. Just how ergot acts in nervous diseases is not known, nor indeed are we likely to know in satisfactory detail the action of any remedy. That ergot contracts the blood-vessels, and thus is useful in local congestion of the brain and spinal cord, is one of the clearly established facts in physiology, and is one of the few definite, solid foundations for therapeutics; but that this effect on the blood-vessels is all that there is in ergot in its action on the body, no philosophical student of nervous diseases would claim. Indeed, this contraction of the blood-vessels must be a result as well as a cause. Behind and beyond all this there is an influence which we cannot analyze. One advantage of ergot is the immediateness of its effects, particularly in spinal hyperæmia. The particular doses of ergot need important modifications in special cases. In some instances, very large quantities of ergotine may be given with benefit and without any harm that I can trace. I give ergot for immediate effects, for sick headaches, and for headaches of other kinds, and for long continued action in spermatorrhœa and various other conditions.

Another of these *Dii majores* of neuro-therapeutics is *arsenic* in its different forms. I use, not only Fowler's solution, but DeVeriangans, with also the English preparation of the chloro-phosphide. Arsenic is a remedy, the effects of which are not, as a rule, felt at once. It needs to be kept up—to be persevered with for many weeks, oftentimes for many months. In some cases, no good comes until the physiological effects have been produced. The great power of arsenic for immediate as well as present effects, has been reecntly impressed upon me by a very remarkable case. A well known physician of New York was under my care for severe neurosis of the stomach, attended with vomiting of all of his food. Though he ate great quantities, he was growing thin and feeble, and he rebelled against nearly every treatment that had been suggested, or, at least, everything that I gave soon lost its effect. I urged him to use arsenic in small doses; at first he was somewhat averse to trying it, and had made up his mind to go to Europe. For a number of months I did not see him, and supposed he had gone to Europe. A short time since he came to my office and reported that he had tried the arsenic as I had recommended, and that the effect had been immediate, and, with but a slight relapse, up to that time permanent. He had gained in flesh, and regained his power to digest food. The remedy, indeed, acted with specific effect upon him.

Another remedy that perhaps will become, if it is not already, one of the major divinities of neurology is *cannabis indica.* This remedy has a reputation of untrustworthiness and unreliability, both of preparation and of action. This reputation it is very fortunately loosing. I find that for some conditions cannabis indica is one of the most trustworthy, most reliable and valuable of remedies. It is one of the drugs, by the proper use of which, the sick headache, for example, has been, within a few years, revolutionized, both for temporary relief at the beginning of an attack and during the attack, and as a permanent cure, provided, its action is maintained for weeks and months. It is one of the most certain and convenient and agreeable of all the preparations used in neuro-therapeutics. Its quick and permanent influence over the symptoms of headache suggests its great value in other conditions allied to sick headache, or from which sick headache springs; and I am accustomed now to use it in the different phases or manifestations of neurasthenia and kindred affection. I use it sometimes alone; sometimes in combination with various tonics and sedatives.

Another remedy, not very widely known, but one the value of which is easily proved, is *cirate caffeine.* Some years since, I called the attention of the profession to the value of this remedy in sick headache as a means of temporary relief at the beginning of an attack; very many physici ns have obtained the same results. I now use this remedy for other symptoms beside sick headache, such as backache—what may be called headache in the back—and malaise, general depression. A disadvantage of this remedy is, that it produces wakefulness, and therefore cannot be taken in the latter part of the day.

Allied to caffeine is *coca*, belonging, indeed, to the same family; indeed, it is the active principle of common coffee, tea, guarana and chocolate. The value of coca as a means of preserving the strength, and abstaining from ordinary food, is erroneously exaggerated in the stock anecdotes that are flitting about our medical literature on this subject; but it has, without doubt, a special and most interesting sustaining and tonic power. It relieves the pain and uneasiness that follow over-exertion, and the peculiar distress that comes from sleepless nights, for which purpose, I may say, caffeine may also be used.

The *zinc* preparations, particularly the bromide, valerianate and oxide are sedatives of very great value in various neurasthenias, and I use them with great freedom. I gave once the zinc combination, including the bromide, the valerianate, the phosphide and the oxide, to a physician who consulted me about a year ago for neurasthenia, resulting from over work in his profession. In a few weeks he reported himself to me to express his gratitude and to testify to the great value of the remedy as a hypnotic as well as a sedative.

*Duboisia*, the new remedy from Australia, is likely to take a minor if not a major place among the resources of the neurologist. Its effect is somewhat like that of atropine, but yet not entirely like it; and, for the symptom of hyperdrosis, seems to be more effective according to experiments that I have made with it.

*Cimicifugin* is a remedy, the value of which in choreic conditions is undeniable, and I am persuaded that its use need not be restricted to those conditions.

There are three other remedies which I use considerably, particularly in renal and bladder complications, and genito-urinal disturbances, viz., the *trailing arbutus*, *eucalyptus*, and *hydrastis*. I believe that these remedies, which I often give in combination, have a tonic power, and are of service even when there is no genito-urinal complication. *Damiana* is a remedy which appears to have a general tonic power in a certain special action upon the lower part of the spinal cord, although I have not yet succeeded in obtaining from it the speedy and immediate effect that has been claimed for it. I use it in combination with other remedies.

Thus far I have spoken mostly of new and unfamiliar remedies; the old medicines, however, must not be forgotten. Much that was valuable in our medication, is as good now as it ever was. When our fathers were consulted for cases of this kind, they treated them, if they treated them at all, with something to act upon the bowels, as cathartics or something of that kind. In this way, they did give some transient relief. I frequently act on this principle, and occasionally administer cathartics to act upon the liver and the bowels. Counter-irritation is certainly a good thing, and cathartics produce counter-irritation of the bowels like a blister on the spine.

*Strychnia* is one of our older remedies, and I use it sometimes alone, but very frequently in combination with other remedies; yet it cannot be used in all cases, for sometimes it has a depressing effect.

*Opium*, in small doses, is excellent for many phases of neurasthenia; and were it not for the danger of forming the opium habit, I should use it more frequently than I do.

*Alcohol* also, in the form of wine, particulary claret and Burgundy, is to be advised in some cases of this kind, but not recklessly, or without reference to the age, character and temperament of the patient. Alcohol is one of the best of our hypnotics in cases where the bromides fail to produce sleep. Where chloral causes severe headache next morning, claret wine, freely used, may produce satisfactory effects without any unpleasant after effects. I do not mention this as a general prescription; I simply say there are cases of which the physician must judge. It has the same objection, however, as opium—that its use may lead to inebriety. In the treatment of nervous cases, it is sometimes necessary to use all of these potent remedies in incredibly and absurdly small doses.

The *mineral acids* are likewise old remedies, but they are good remedies. Dilute nitro muriatic acid, either alone or combined with the vegetable bitters, I use in different forms of nervous exhaustion, especially where the urine is over loaded, as it often is, with oxalates and urates.

Of *cod-liver oil*, I may say that it probably does more for the nervous than it does for the consumptive. Oil and fats, like cream and butter, are brain food, and if used judiciously, as the stomach can bear them, act both as food and as medicine. The oil I use generally in the form of emulsion, and I use it with great freedom.

Of *phosphates*, this can be said: that they belong to the list of over-praised and over-used remedies. There is a fashion for phosphates just now, and when men become neurasthenic, they think they are on the road to health if they take some of the phosphates or phosphites. Now, these phosphates and phosphoruses and phosphites, are good remedies in nervous troubles; but if they had anything like the specific power claimed for them, there would be little need for treating these cases; most of the patients that I see have taken them in abundance. All these stock remedies have a certain power which, in very many cases, they soon expend —they reach the limit of effect, beyond which they cannot be forced.

Another new remedy, or comparatively new to this country, is *koumiss*—fermented milk. The power of this remedy to produce sleep is very great, and very satisfactory. It is a means of nourishing the body without disturbing or even using the stomach to any very great degree. Koumiss is really digested milk, and is absorbed and taken up into the system without any strain upon the digestive apparatus. My friend, Dr. Brush, who has given attention to the study of this subject, tells me that from experiments which he made sometime since, it was pretty clearly proved that the alcohol which the koumiss contains was used up in the system and not eliminated. I am persuaded that the use of koumiss in the future is to be very widely extended for all conditions where nutrition is difficult—not only in adults, but in children. The one disadvantage of koumiss in some cases—that it constipates the bowels—is to be met by laxatives.

Another very old remedy, but as good as it is old, where it is properly used, is counter-irritation, which I employ both in the form of *actual cautery*, and galvanic cautery, and very small blisters, so small and so arranged as to cause very little annoyance. Counter-irritation in the hands of those who really understand how to use it without abusing it, is one of the three or four major remedies of neuro-therapeutics.

There is among the people, and even in the profession, a prevalent notion that the application of the actual cautery is a very painful procedure. This false idea has been fostered with the public on account of the supposed sufferings of certain prominent persons, like Charles Sumner, and Clara Morris, the actress, from this treatment. The lectures of Brown-Sequard, referring to this subject, assisted in confirming this impression, and the newspaper accounts, in every possible way, have stimulated and strengthened the belief that it requires the courage of a hero, to submit without etherization, to the operations of the actual cautery. The idea of fire is always troublesome to the human mind; the idea of hell is associated with infinite burning. All these factors together have made it difficult for physicians, in modern times especially, to resort to the use of the cautery as often as it might be well to do. The real scientific truth on this matter is, that the actual cautery, as it can be used, and is used by those who understand it, is not specially painful, even to the most delicate women. The pain is in the idea of the thing—in the expectation, and not in the burning. Any one who has had a sensitive tooth filled, has suffered ten times more than one who has submitted to a cautery operation, if properly performed. I speak of this point particularly, because the cautery is an agent of such great therapeutical power. This mode of treatment, like the blisters already referred to, must be, and now can be, modified and adapted to the sensitive modern constitution. It is one of the great remedies that stands the test of time and large experience.

*Hydro-therapeutics* in the form of bags of hot and cold water, the Russian and Turkish baths, and alternate applications of hot and cold is, in skillful and judicious hands, a great remedy for functional nervous diseases. The difficulty is that men prescribe these things for themselves, and use them for themselves and by themselves, and often get evil when they might get good. Every remedy that is good for anything, may, when improperly applied, do harm; but harm in these cases is not due to the remedy, but to the one who uses it.

It is impossible to speak of the treatment of this class of troubles without referring to the *bromides* of potassium, and sodium and lime and lithium. Bromides may not be classed among the old remedies. Their great value in epilepsy has long been known. They are not, however, so well understood in other nervous diseases of a functional character. The bromides may be used in large doses, frequently repeated until the powerful sedative effect is produced, even when there is no sleeplessness; those who use the bromides in this way must know where to stop or to reduce the dose.

There are a few general principles of treatment of which I will speak. First of all, the proper use of *rest* and *work* in the treatment of nervous disease. About a month ago, a patient with ataxy came to me from a distant city in the West; I said to him, "you have left behind you a better doctor than you can find here." He asked, "Who?" I said "*rest!*" I prescribed it for him, and put him to bed. He had been accustomed to take excessive exercise—at least, far more than was good. The next day, another gentleman came, also from a distant city of the West, with the history of a certain form of cerebrasthenia—brain exhaustion—without any myelasthenia, or spinal exhaustion—and of a type that would be benefited rather than injured by a degree of mental and physical activity. He had felt disheartened and thought there was little for him to do in this world. He was of about middle life, and I told him that he probably was no more than "half-way home," and so far as the disease was concerned, he might live and be active for thirty or forty years longer. When he returned, I said to him, "you have come a long distance to consult me, but you left at home a better physician than you can get here." He asked, "Who?" I said "*work;* work I prescribe for you. Take that in connection with all your medicine and you will recover." These two cases make clear the opposite methods of treatment.

A second and general suggestion is, that of a stopping treatment or suspending it at times. Suspending treatment has a positive effect upon the system. Oftentimes it makes a direct impression, which may be better than continuous treatment. A friend of mine, formerly a sea captain, states that when sleeping in his cabin at night, if the sentinel walking the deck above him stopped, it would always wake him. The sudden sensation of nervous activity, like a jar upon the nerves, aroused him from his slumber. I find that patients sometimes do better—make more decided progress—in these intervals of treatment than while the most active measures are being used. Patients sometimes imagine this a proof of the valuelessness of the medicines; but it is in reality a proof of their power. It has been said that success in life depends largely upon knowing just where to stop. In the practice of medicine, this maxim is certainly sound; and to know where to stop, to let up, to modify the treatment, is one of the best tests of medical skill.

The third general suggestion is, that in the treatment of nervous diseases, we should study with all our energy the psychology of our patients; we must make a diagnosis of the intellectual character as well as of the disease before we can make a prognosis or adopt a plan of treatment. There are those whose minds are so organized, which lack some qualities and have excesses of others—usually a preponderance of the emotional, with a deficiency of the higher intellectual qualities—that they act badly under any treatment, however wise. Some patients take a pleasure in their distresses; it would be cruel to cure them; their pains are their possessions. Any man wishing to make them well would be no better than a thief or a robber. There are those whose chief felicity in life consists in doctoring and being doctored, and to whom the removal of their bodily ills would be like the death of long cherished friends. When such persons come under your care, you cannot expect any treatment to be as successful as with those strong and active intellects, who understate rather than magnify their troubles, and are resolutely determined to get well.—*Virginia Medical Monthly.*

